# The Cyclical Battle of Insomnia and Mental Health Impairment in Firefighters: A Narrative Review

**DOI:** 10.3390/jcm13082169

**Published:** 2024-04-09

**Authors:** Angelia M. Holland-Winkler, Daniel R. Greene, Tiffany J. Oberther

**Affiliations:** Department of Kinesiology, Augusta University, 3109 Wrightsboro Road, Augusta, GA 30909, USA; dagreene@augusta.edu (D.R.G.); toberther@augusta.edu (T.J.O.)

**Keywords:** sleep quality, first responders, PTSD, anxiety, depression, emotional regulation, substance abuse, suicide

## Abstract

The occupational requirements of full-time non-administrative firefighters include shift-work schedules and chronic exposure to alerting emergency alarms, hazardous working conditions, and psychologically traumatic events that they must attend and respond to. These compiling and enduring aspects of the career increase the firefighter’s risk for insomnia and mental health conditions compared to the general population. Poor sleep quality and mental health impairments are known to coincide with and contribute to the symptom severity of one another. Thus, it is important to determine approaches that may improve sleep and/or mental health specifically for firefighters, as their occupation varies in many aspects from any other occupation. This review will discuss symptoms of insomnia and mental health conditions such as PTSD, anxiety, depression, substance abuse, and suicide in firefighters. The influencing factors of sleep and mental health will be examined including anxiety sensitivity, emotional regulation, and distress tolerance. Current sleep and mental health interventions specific to full-time firefighters are limited in number; however, the existing experimental studies will be outlined. Lastly, this review will provide support for exploring exercise as a possible intervention that may benefit the sleep and mental health of this population.

## 1. Introduction

The occupational demands of firefighters are often stressful, tense, and volatile [1]. They must be prepared to respond to a wide range of unpredictable and often hazardous emergencies. Although firefighters are thoroughly trained to be safe in dangerous situations, they are still highly susceptible to injury. They also face psychological trauma as they are the first to respond to suffering individuals (i.e., a child in trouble or suffering) [2,3,4]. At the fire station, they are exposed to loud shrilling emergency alerting alarms, during both the day and night, which frequently and rapidly activate their sympathetic nervous system [5,6]. These physical and psychological stressors encountered every working shift have been shown to lead to increased risk for major health conditions such as cardiovascular disease, cancer, and depression [7,8,9].

In addition to these stressors, most firefighters are shift-workers. Shift schedules vary across fire departments; fire departments may adopt a 24 h on/48 h off or 48 h on/96 h off schedule, while others may adopt a more complex schedule like the Kelly schedule which consists of 24 h on/24 h off/24 h on/24 h off/24 h on/96 h off [10,11]. Some firefighters may even work shifts that last as long as 72 h [12]. During working shifts, firefighters are expected to be on call and respond immediately to emergency alarms [5]. This required alertness significantly impacts sleep while on shift and may also impact sleep patterns off shift as well, leading to sleep deprivations, severe circadian dysrhythmias, and possibly chronically impaired sleep patterns [7,13]. The internal circadian rhythm synchronizes with external environmental cues including light, physical activity, and melatonin secretion with light exposure being the most dominant regulator [14]. The misalignment of the circadian rhythm with the 24 h outside environment may lead to a circadian rhythm disorder which negatively affects sleep and may lead to health conditions including metabolic, cognitive, cardiovascular, and gastrointestinal impairments [15].

Sleep is vitally important to both physical and mental health [16]. Sleep recommendations include seven to nine hours of sleep for adults, with continuous cycles of rapid eye movement (REM) and nonrapid eye movement (NREM) sleep over the course of the total sleep period [17]. Each cycle of REM and NREM occurs every 90–120 min, with it taking at least one hour to reach NREM sleep [18]. REM sleep occurs after slow-wave sleep and allows for emotional memory processing, while NREM sleep is required for optimal wakefulness and cognitive function the following day [19,20,21,22,23]. Seven to nine hours of sleep is recommended to allow for three to six REM/NREM cycles per night [24]. This consistency of sufficient sleep patterns helps regulate circadian rhythm which in turn promotes sufficient sleep patterns and health.

Optimal wakefulness and cognitive function occurring from NREM sleep are required for the intense physical and mental obligations of firefighting [25]. In addition, emotional memory processing and thought processing that occurs through REM sleep is valuable to the long-term psychological and even physical health of firefighters [26,27,28]. However, firefighters commonly suffer from both insomnia and mental health conditions due to the nature of their occupation. Mental health conditions common in firefighters include post-traumatic stress disorder (PTSD), depression, anxiety, and substance abuse which may cause a greater risk for suicidal thoughts and attempts [7,29,30,31]. Many studies have sought to determine the relationship between insomnia and mental health as well as interventions that may improve these conditions in firefighters [10,30]. This narrative review will discuss the prevalence, mechanisms, and relationship of insomnia and mental health disorders in firefighters as well as interventions that have been utilized to improve these conditions.

## 2. Insomnia and Firefighters

### 2.1. Insomnia Evaluation

In general, insomnia occurs when someone has difficulty sleeping [32]. Diagnosed clinical insomnia can be categorized as acute insomnia and/or chronic insomnia. Acute insomnia occurs when a person has difficulty going to sleep and/or staying asleep at least three days per week for a period of one week to three months [33]. There are multiple types of acute insomnia such as transient and sub-chronic insomnia, but for the scope of this review, these sub-categories of acute insomnia will not be discussed in detail [33]. A diagnosis of chronic insomnia may occur if a person has difficulty going to and/or staying asleep at least three days per week for at least three months, despite having sufficient time to sleep. Additional criteria for chronic insomnia include significant daytime impairment or distress [33,34,35].

Schutte-Rodin et al. [36] describe the guidelines for a clinical evaluation of insomnia which include a thorough sleep history and a detailed medical, substance, and psychiatric history. It is recommended for the evaluation to consist of the following subjective reports or measures: medical and psychiatric questionnaire, subjective sleep assessment (i.e., Epworth Sleepiness Scale), and a two-week sleep log. Polysomnography and actigraphy are additional objective assessments that may provide beneficial data for further insomnia evaluation [36].

Population-based epidemiological studies over the past few decades have reported variable rates of insomnia in general populations, but the consensus from those studies is that 30–36% of adults report having at least one nighttime sleep-related symptom of insomnia, while 10–15% of the population has at least one nighttime sleep-related and one daytime fatigue-related symptom of insomnia [26,37]. However, when more rigorous insomnia criteria are used to determine insomnia rates (i.e., diagnostic criteria from the Diagnostic and Statistical Manual of Mental Disorders IV; DSM IV), the prevalence in adults decreases to 6–10% [38,39]. It is therefore difficult to compare insomnia rates in various populations when different criteria are utilized.

### 2.2. Sleep Measures in Firefighter Studies

The terms sleep deprivation, sleep disorder, disturbed sleep patterns, clinical insomnia, and poor sleep quality are commonly used in firefighter-based sleep studies. Sleep patterns in firefighters are often assessed with the Pittsburgh Sleep Quality Index (PSQI), a validated subjective questionnaire that measures sleep quality and disturbances over a one-month period. It includes 19 items to then provide scores for seven components of sleep quality on a “0” (“best sleep quality”)-to-“3” (“worst sleep quality”) scale; those seven components include subjective sleep quality, sleep latency, sleep duration, habitual sleep efficiency, sleep disturbances, the use of sleep medication, and daytime dysfunction [40]. The seven scores are summed, and a score of 5 or less indicates good sleep quality, and a score of greater than 5 indicates poor sleep quality.

Another subjective sleep quality questionnaire frequently used in this population is the Epworth Sleepiness Scale (ESS) which differs from the PSQI as it assesses the likelihood of falling asleep in various daytime scenarios. The ESS asks participants to indicate, on a 4-point Likert scale, how likely they would be to fall asleep with “0” indicating “no chance of dozing” and “3” indicating a “high chance of dozing”. A total score of 0–9 indicates that “you are not abnormally sleepy”, a score of 10–15 indicates that “you may be excessively sleepy depending on the situation and you may want to consider seeking medical attention”, and a score of 16–24 indicates that “you are excessively sleepy and should consider seeking medical attention”. When compared to other clinical measures of sleep quality, it was found valid and reliable [41]. Many studies use both the PSQI and ESS to assess nocturnal sleep difficulties and daytime sleepiness together. 

Lastly, the insomnia severity index (ISI) is another validated subjective questionnaire used in this population to measure insomnia severity [42]. The ISI consists of seven sleep-related questions rated on a five-point Likert scale with “0” corresponding to “no problem” and “4” corresponding to a “very severe problem”. A score of 15 or higher indicates clinical insomnia with a score of 15–21 being categorized as “moderate insomnia” and 22–28 being categorized as “severe insomnia”. Multiple studies have utilized objective sleep assessments such as an actigraphy motion logger which may be worn for periods of days to weeks to detect sleep patterns.

### 2.3. Poor Sleep Prevalence in Firefighters

The prevalence of poor sleep quality in firefighters has been reported by various studies and differs based on country and sleep quality measures. In a large study by Horn et al. [30], 880 current and retired United States firefighters completed a web-based ISI and 52.7% reported clinically significant insomnia symptoms with 19.2% reporting nightmare problems. Cary et al. [7] showed that 59% of full-time firefighters in a metropolitan area of the United States reported sleep deprivation which was measured with the PSQI and ESS questionnaires as well as a 3-day actigraphy motion logger. They considered the firefighter to have a disturbed sleep pattern if they had at least two of the following sleep issues: sleep duration <6 h, sleep efficiency <85%, sleep latency >30 min, or wake after sleep onset >30 min. Billings et al. [11] assessed sleep quality in 109 full-time shift-working firefighters in the United States using the PSQI and found that 73% reported poor sleep quality; those that worked a second job had significantly poorer sleep quality than those who did not. Thus, in the United States, the prevalence of poor sleep quality in firefighters ranges from 52 to 73% in studies that include over 100 participants.

Sleep quality has been assessed in firefighters outside of the United States with similar results. Mehrdad et al. [43] assessed sleep quality in 427 Iranian firefighters using the Persian version of the PSQI. They received an 88.7% response rate with 69.9% reporting poor sleep quality. Lim et al. [44] assessed the sleep quality of 657 full-time firefighters in a metropolitan area of South Korea using the PSQI. In total, 48.7% reported poor sleep quality; however, shift-workers reported significantly higher levels of poor sleep quality (51.6%) than the non-shift-workers (38.5%). In a large study by Cramm et al. [10], 69% of 1217 volunteer and full-time Canadian firefighters self-reported their sleep quality as “fair” to “very poor”, and 21.3% were found to have clinical insomnia based on the ISI scores. Thus, the prevalence of poor sleep quality ranges depending on the assessment and the working schedule of the firefighters but is typically higher than the general population of the area [10,11,26,45].

The incidence of chronic insomnia in firefighters is difficult to accurately determine based on the results from most experimental or observational sleep studies in this population as the sleep assessments are typically based on a one-month period, whereas a diagnosis of chronic insomnia requires a minimum of a three-month assessment or report of sleep difficulty. However, since firefighters are more likely to have sleep impairments than the general population, it can be assumed that the nature of their occupation may be the overarching factor resulting in the poor sleep quality found with the one-month assessments. Thus, if the firefighter has a history of employment longer than three months, it could be assumed that the one-month assessment of poor sleep quality would occur throughout employment and be considered chronic. However, this is an assumption and needs further exploration.

## 3. Mental Health and Firefighters

Firefighters are regularly exposed to significant physical, emotional, and psychological challenges. As part of their daily routine, every call exposes them to a potentially traumatic event. As such, it comes with little surprise that firefighters often report elevated prevalence rates associated with mental health disorders. A recent systematic review examining the mental health of firefighters relative to the general population has concluded firefighters are significantly more likely to experience PTSD, depression, and anxiety [46]. Wolffe et al. [31] assessed the overall prevalence of mental health disorders among UK firefighters relative to the general English population. Approximately 12% of firefighters reported anxiety, 10% reported depression, and 5% reported PTSD. In contrast, approximately 6% of the general UK population reported anxiety, 3% reported depression, and just under 5% reported PTSD [31].

### 3.1. Anxiety

According to the Diagnostic and Statistical Manual of Mental Disorder (DSM-5), anxiety is a normal response to stress, while an anxiety disorder is associated with intense feelings of excessive worry and apprehension. Additionally, the DSM-5 describes an anxiety disorder as a chronic condition in which individuals experience symptoms on most days (i.e., more days than not) over the previous 6 months [47]. While anxiety is inherently a negative stress response, not all stress negatively impacts you. There are two distinct responses individuals experience in the presence of stress. Individuals can feel motivated, excited, and approach a stressful situation as a challenge to overcome. This is referred to as eustress or positive stress and can be beneficial for overall health and wellness [48]. However, stress can promote feelings of anxiety, discomfort, and decrease motivation. This type of stress is negative and referred to as distress [48]. While there are specific stressors that typically promote eustress (e.g., exercise/physical activity) or distress (e.g., traumatic events) there are individual characteristics that act to protect one from significant distress such as anxiety sensitivity and emotional regulation [49]. As firefighters are naturally exposed to potentially traumatic events, these individual characteristics could be a significant factor concerning mental health in this population.

As mentioned above, firefighters are exposed to extremely stressful situations and often experience increased anxiety relative to the general population. To highlight this, a recent report identified a career as a firefighter to be the second most stressful job in the United States [50]. Wolffe et al. [31] reported that firefighters are more than twice as likely to experience anxiety relative to the general population. The Health Hazard Evaluation Program reported that 53% of firefighters experienced symptoms of Generalized Anxiety Disorder (GAD) [51]. A recent meta-analysis assessed 8096 first responders and reported 60% met criteria for mild anxiety, 27% met criteria for moderate anxiety, and 14% met criteria for severe anxiety [52]. While the symptoms of anxiety can be severe and debilitating on their own, individuals living with higher levels of anxiety are more susceptible to other mental health conditions. It has been well established that firefighters experience significant occupational stress, and reports indicate that occupational stress is a significant predictor of PTSD symptomology in firefighters [53]. Occupational stress in firefighters has been linked to numerous other mental health concerns. First, as mentioned above, individuals can have a more positive (i.e., eustress) or more negative (i.e., distress) response to external stressors. While occupational stress is inherently negative, increased occupational stress in firefighters has been correlated to increased PTSD symptom severity, depression, and anxiety [54,55]. Firefighters with higher occupational stress are more likely to experience severe mental health disorders, and this relationship is further exacerbated by disturbances in sleep.

According to the UK Firefighter Contamination Survey, 46% of firefighters self-reported their mental health to be a significant factor impacting their sleep disturbance [31]. Yook et al. [56] examined the relationship between occupational stress and sleep quality in 705 male firefighters. The firefighters were evenly divided into a low stress, medium stress, or high stress tertile group. Firefighters in the high stress group experienced significantly worse outcomes in subjective sleep quality, sleep latency, sleep duration, habitual sleep efficiency, sleep disturbances, the use of sleeping medication, and daytime dysfunction relative to both low and medium stress groups. Further, firefighters in the medium stress group reported significantly worse outcomes in subjective sleep quality, sleep disturbances, and daytime dysfunction relative to the low stress group [56]. While the prevalence of anxiety has been linked to disturbed sleep, this is a reciprocal relationship. Data from the National Canadian Mental Health Survey indicated firefighters with insomnia to be 7.15 times more likely to suffer from GAD relative to firefighters without insomnia [10]. Simply by examining anxiety, the cyclical relationship between mental health conditions and insomnia can be seen in firefighters.

### 3.2. Depression

Another leading mental health concern for firefighters is depression. According to DSM-5 criteria, an individual must experience five or more major depressive disorder (MDD) symptoms during the same two-week period to be diagnosed with MDD. These symptoms include a depressed mood, a diminished interest in activities that used to be pleasurable, a significant change in body weight without apparent effort, insomnia, significant fatigue, feelings of worthlessness, decreased concentration, suicidal ideation, and psychomotor agitation [47]. While estimates vary, it is generally accepted that firefighters experience elevated depression and MDD relative to the general population. A large study estimates the 12-month depression prevalence to be between 5.5 and 6.0% in the general population [57] where estimates are significantly higher in firefighter populations (e.g., 40% [30]). Within the firefighter population, there are significant risk factors associated with experiencing greater levels of depression. Rescue and disaster workers who were exposed to a disaster were significantly more likely to experience depression at 7 and 13 months following the event relative to rescue and disaster workers who were not exposed to a similar event [58]. Specifically, 21.7% of the exposed workers experienced depression, while 12.6% of non-exposed workers experienced similar feelings of depression 13 months after the event [58].

While it is clear that firefighters are more likely to experience elevated depression and MDD relative to the general population, it is equally important to explore specific comorbidities linked to depression within this population. Individuals living with MDD have reported a significant increase in other mental health disorders including anxiety [59], alcohol and drug use [60], and suicidal ideation/attempts [61]. These relationships are present and often exacerbated in firefighters. In a sample of 169 firefighters exposed to a traumatic event, 53.3% reported depression and 57% met the full DSM-IV criteria for PTSD. Further, depression and PTSD were significantly correlated [62]. In addition to other mental health disorders, depression has been linked to other psychological disturbances.

There is a strong amount of evidence linking insomnia as a risk factor for depression [26]. Even non-depressed individuals have a twofold risk for developing depression if they have insomnia compared to people that do not suffer from sleep difficulties [63]. Firefighters suffering from disturbed sleep were shown to be twice as likely to have anxiety or depression. Further, firefighters with sleeping problems were over four times as likely to experience a mental health concern [31]. Cramm et al. [10] assessed 1217 firefighters from the National Canadian Mental Health Survey and reported firefighters with insomnia to be 7.91 times more likely to experience MDD than firefighters without insomnia. Similarly, Horn et al. [30] assessed 880 United States firefighters with cross-sectional surveys and found that 39.6% had clinically significant symptoms of depression, 52.7% had insomnia symptoms, and 19.2% had nightmares; they suggested that insomnia may increase the risk for depression through the impairment of emotional regulation (described below).

### 3.3. PTSD

According to the DSM-5 criteria, an individual is likely to have PTSD following exposure to a traumatic event if they exhibit symptoms of re-experiencing, avoidance, negative alterations in cognitions and mood, and hyperarousal [47]. Firefighters are at a significantly higher risk of experiencing PTSD symptoms. Specific studies have estimated the rate of PTSD symptoms to affect approximately 17% of Canadian firefighters [29], 18% of German firefighters [64], and 22% of American firefighters [29]. In a sample of 3289 firefighters, 13% (i.e., 432) reported symptoms of PTSD [65], and a recent review reported the PTSD prevalence in firefighters to be as high as 57% [66]. This is striking as estimates of the PTSD prevalence in civilian populations have been estimated as low as 2.3–2.9% [67]. Firefighters with PTSD symptoms were significantly more likely to suffer from work-related injuries, chronic musculoskeletal disorders, burnout, and poor physical condition [65]. Further, Fullerton et al. [58] examined disaster workers who were exposed to a traumatic event relative to comparison workers who were not exposed. Their results indicate that 16.7% of exposed workers reported PTSD while only 1.9% of non-exposed workers reported PTSD 13 months following the incident.

PTSD is a trauma- and stress-related disorder, but prior to 2013, PTSD was classified as an anxiety disorder [47]. Thus, PTSD shares numerous risk factors and comorbidities with other mental health conditions. According to data from the National Comorbidity Survey, of those diagnosed with PTSD, 54% reported having a major depressive episode, 28% reported having GAD, 28% reported alcohol abuse/dependence, and over 43% identified as having three or more other diagnosed conditions [68]. Specifically in firefighters, PTSD symptom severity has been associated with numerous other mental health concerns. Bing-Canar et al. [69] assessed 632 trauma-exposed firefighters and found PTSD symptoms to significantly predict suicide risk. A recent systematic review has identified anxiety, depression, work-related injuries, and chronic musculoskeletal disorders as significant comorbidities that predict PTSD prevalence among firefighters [70].

Insomnia and other sleep disturbances are diagnostic symptoms of PTSD and not easily treated. Sleep disturbances mediate PTSD, leaving suffering individuals to function poorly during the day in addition to promoting poorer PTSD-related clinical outcomes such as increased alcohol use, poorer physical and mental health, and reduced overall quality of life [28]. With sleep disturbances being resistant to first-line treatments for PTSD (i.e., pharmacotherapy [71] and cognitive behavioral therapy [72]), firefighters suffering from PTSD and insomnia may need multiple forms of treatment; however, this needs further exploration.

### 3.4. Obsessive–Compulsive Disorder (OCD)

While the prevalence of OCD is not as widespread as other mental health concerns, firefighters are at an increased risk for living with OCD. According to a large survey of 10,649 firefighters, 1.6% reported having OCD [31]. Despite the relatively low prevalence of OCD in firefighters, it is important to reference the general population. A large epidemiological study of 25,180 individuals from Iran found the prevalence of OCD to be 1.8%, with females significantly more likely to experience OCD relative to males [73]. Further, a recent meta-analysis highlights the notion that women are 1.6 times more likely to experience OCD relative to men [74]. Given the relatively similar prevalence rates of OCD in firefighters and the general population, the sex differences highlight an interesting notion. Specifically, over 95% of all US firefighters between 2011 and 2015 were male [75]. Therefore, it is reasonable to conclude that the 1.6% prevalence rate of OCD in predominantly male firefighters is significantly greater than the average OCD rate reported by men. Specifically, the prevalence of OCD has been estimated at 0.7% and 2.8% for males and females, respectively [73].

OCD has been linked to numerous other mental health concerns. Hofer et al. found strong evidence linking prior OCD to an increased risk of GAD and social phobias [76]. Individuals living with OCD have a significantly greater risk of suicidal thoughts and actions. The presence of depression further exacerbates the increased risk of suicidal ideations [77]. Specifically in firefighters, there is evidence that the demands of the job can lead to an increased risk in developing OCD or OCD symptoms [78].

### 3.5. Suicide

Suicide is another mental health concern that is of paramount importance with respect to firefighters. Overall, first responders have been identified as a population at increased risk for numerous mental health concerns including suicide [79]. Within the general population, 13.5% have reported suicidal thoughts, 3.9% have reported suicidal plans, and 4.6% have reported a suicide attempt [80]. While these numbers are striking, firefighters have reported significantly greater suicide behaviors. Specifically, 46.8% of firefighters have reported suicidal thoughts, 19.2% have reported making a suicide plan, and 15.5% have experienced a suicidal action [81]. Suicide takes the lives of 800,000 people each year [82]. While it is important to highlight the increased risk firefighters face with respect to suicidal thoughts and actions, it is equally important to understand other risk factors associated with suicide.

Suicide has been associated with numerous other mental health outcomes. Survey results indicate both PTSD and GAD to be significant predictors for suicidal ideation and attempts [83] in a large sample of adults from the general population. This relationship has also been shown in firefighters. Firefighters with greater PTSD symptom severity [84] and levels of depression [85] reported a significantly greater risk of suicide. Suicidal ideation and attempts have also been linked to other psychological factors such as sleep. A National Comorbidity Survey was filled out by 8098 adults in the United States and assessed to determine the association between sleep, mental disorders, suicidal ideation, and suicide attempts over the past 12 months and lifetime. The results demonstrated that short sleep increases the chances of suicidal ideation and suicide attempts, regardless of mental conditions. Furthermore, suicidal ideation and short sleep was shown to increase the odds of a suicide attempt and was additionally related to the presence of substance use disorders [86]. Vargos de Barros et al. [16] assessed six mental health conditions in 303 Brazilian firefighters which included stress, depression, anxiety, suicidal ideation, alcohol use disorders, and general health with subscales of a desire for death, psychosomatic problems, and sleep disturbances. Of the 303 firefighters, 51% had sleep disturbances which were significantly related to the presence of psychological distress and psychosomatic alterations. Suicidal ideation was found in 15% of the firefighters and unhealthy alcohol use in 31%; both conditions trended toward a significant association with sleep disturbances (*p* < 0.085). A case study described an older man with poor sleep quality, excessive daytime sleepiness, a depressed mood, and suicidal ideation with active suicide plans that was treated for sleep apnea without antidepressant medication [87]. He responded well to the sleep apnea treatment (nCAP) and his suicidal ideation and depression resolved, which demonstrates the need to treat sleep conditions to improve the outcomes of other likely associated mental conditions.

### 3.6. Substance Abuse

Firefighters have been identified as a population at significantly greater risk for substance abuse. A recent national survey of 674 firefighters concluded over 48% displayed heavy drinking behaviors and over 43% met the criteria for binge drinking [88]. Relative to the general population, these numbers are significantly elevated. According to a recent meta-analysis including six national surveys, about 25% of the United States’ adult population reported binge drinking behavior [89]. Interestingly, recent reports indicate the general female population displays significantly lower levels of binge drinking behavior (i.e., about 13.5% [90]). Thus, Haddock et al. [90] assessed drinking behavior in a large sample of female firefighters (N = 1913). Their results indicate about 40% of female firefighters reported binge drinking. Even though female firefighters only account for roughly 5% of the firefighter population, they also experience a significant increase in substance abuse behavior [91].

Firefighters are also at an increased risk for using other psychoactive substances. In a sample of 112 firefighters, 58% displayed binge drinking behavior, 20% identified as nicotine users, and 5% met criteria for caffeine overuse (i.e., >700 mg/day) [7]. While less is known about specific drug use among firefighters, there is some evidence of anxiolytic drug use. Specifically, in a recent survey of 711 military firefighters, the anxiolytic drug use was 9.9% [92]. Another survey on firefighters and psychoactive substance use included questionnaires on tobacco, alcohol, prescribed medication, and illicit drug use. Among the 168 male firefighters included, 89.9% reported alcohol consumption, 38.1% reported smoking tobacco, 2.4% reported taking prescribed medications, and 1.2% reported illicit drug use [93]. While it is possible firefighters are not accurately self-reporting medication and drug use, the main area of concern among this population appears to be alcohol use. While substance abuse is a very important mental health concern, it has also been linked to other mental health conditions such as PTSD [94], depression [7], and sleep quality [7] in firefighters. As mentioned above, firefighters can be under extreme occupational stress and might seek external coping mechanisms. Alcohol consumption has been identified as one of the most prominent coping strategies used by firefighters in response to occupational stress [95,96]. Further, alcohol consumption has been associated with suicide risk among firefighters [69]. To explore this phenomenon, Martin et al. [97] examined the impact of alcohol dependence on suicide risk in 2883 male firefighters. Their results indicated a significant association between alcohol dependance and suicide risk in firefighters. Additionally, they found evidence linking alcohol dependance to increases in depression, which further predicted suicide risk factors [97]. This highlights the cyclical nature of mental health disorders among firefighters.

Alcohol is a substance that causes sleepiness and may help non-alcoholic healthy individuals fall asleep faster and have better NREM sleep; however, it may disrupt sleep during the second half of the night. Both periods of binge drinking and alcohol abstinence have been shown to disrupt sleep [98]. Smith et al. [99] assessed sleep, mental health, and alcohol use in 639 urban career firefighters and found a significant positive correlation between sleep disturbance severity and alcohol use severity as well as alcohol use for coping reasons. In addition, there was a significant positive correlation between PTSD severity and alcohol use severity and alcohol use for coping reasons which was even stronger when sleep disturbances were high in the firefighters. The cyclical nature of these impairments underlines the importance of finding treatment options that may improve the health of more than one condition.

### 3.7. Implications

Based on the evidence above, mental health conditions such as anxiety, depression, PTSD, substance abuse, and suicide risk factors are often comorbidities of each other and with sleep disturbance. However, it is important to note that one is not caused by the other. That is, while individuals living with MDD are more likely to be diagnosed with GAD, both MDD and GAD have significant and independent impacts on mental health [100]. Further, Elhai et al. assessed the overlapping symptoms associated with PTSD, anxiety, and other mood disorders. After controlling for overlapping symptoms, the overall PTSD prevalence only decreased by 0.39%, from 6.81 to 6.42% [68]. This suggests that symptoms from a specific mental health condition will not cause another mental health condition but rather increase the susceptibility to separate, independent comorbidities. Taken together, this highlights the cyclical battle firefighters face with mental health and insomnia.

## 4. Factors Influencing Sleep and Mental Health

### 4.1. Anxiety Sensitivity

While firefighters are exposed to traumatic events that increase their risk of experiencing negative mental health outcomes, there may be specific traits that protect against this. One individual trait that may influence mental health outcomes following exposure to a traumatic event is an individual’s fear of arousal-related situations (i.e., anxiety sensitivity). According to Taylor et al. [101], anxiety sensitivity can be accessed via the 18-item, self-report anxiety sensitivity index (ASI-3). The ASI-3 is a valid assessment of general (i.e., global) anxiety sensitivity and physical (i.e., fear induced by increased heart rate), cognitive (i.e., fear induced by concentration difficulties), and social (i.e., fear induced by being observed in anxiety-producing situations) concerns [101]. General anxiety sensitivity has been shown to significantly affect the symptoms of PTSD, panic disorder, depression, and social anxiety [49]. Stanley et al. [102] assessed 254 female firefighters and found anxiety sensitivity to be a significant predictor of suicide risk.

Although anxiety sensitivity has been shown to predict symptoms of mental health in firefighters, the relationship is reciprocal. Specifically, PTSD symptom severity in female firefighters significantly predicted the individual difference factor of anxiety sensitivity [102]. This has potentially important implications for firefighters as both a screening tool and a specific target for interventions. Given that firefighters with elevated anxiety sensitivity are at an increased risk of experiencing PTSD, depression, panic, and social anxiety, it follows that interventions to decrease anxiety sensitivity would decrease these risk factors. Alternatively, interventions that decrease specific risk factors of anxiety sensitivity (e.g., PTSD symptoms) could have large implications on the overall mental health of firefighters.

Insomnia and anxiety sensitivity have been strongly linked in multiple studies [103,104,105]. Kim et al. [106] assessed 95 mentally healthy adults in the general population of Korea to determine the relationship between insomnia severity and anxiety sensitivity as well as other mental health conditions. They found that insomnia severity was significantly correlated with anxiety sensitivity, as well as depression, and anxiety. In a randomized clinical trial, the efficacy of treating anxiety sensitivity on reducing insomnia symptoms was investigated in 151 patients. The findings demonstrated that computerized treatment for anxiety sensitivity resulted in significant reductions in insomnia symptoms at three- and six-month follow-up periods, even when controlling for reductions in depression and anxiety [107].

### 4.2. Emotional Regulation and Emotional Intelligence

Emotional regulation and intelligence have also been examined as potential mediating factors explaining the relationship between mental health and insomnia. Emotional regulation is an individual difference trait that explains, to some degree, the level of control an individual has over their emotions. Specifically, in a given situation, emotional regulation can affect the intensity, duration, and type of emotion exhibited [108]. Individuals living with significant mental health symptoms associated with depression, anxiety, and/or PTSD (to name a few) may experience an impairment in emotion regulation. Thus, individuals are at an increased risk of encountering negative emotional states and are less equipped to effectively cope with them, leading to decreased sleep quality and insomnia [30]. On the other hand, individuals with higher emotional intelligence display an increase in psychological well-being [109]. Emotional intelligence highlights an individual’s ability to recognize their emotions and the emotions of those around them, label these emotions effectively, and use this information to make rational decisions [110,111]. As established above, firefighters are exposed to significantly greater levels of occupational stress. Emotional intelligence has been shown to indirectly influence perceived stress [112], thus individuals with higher emotional intelligence may experience a blunted stress response or decreased distress during traumatic events.

Daily emotional distress is regulated during REM sleep as limbic activation during REM sleep reactivates emotionally distressing events, associates them with previous occurrences, and then processes them [113]. Restless or fragmented REM sleep interferes with the process of distress resolution thereby contributing to a state of chronic hyperarousal, which is a primary symptom of insomnia [114,115]. Interestingly, Wassing et al. [27] demonstrated that maladaptive sleep periods associated with insomnia may worsen the traumatic exposures compared to those without insomnia; thus, instead of sleep resolving emotional distress, the maladaptive sleep periods increased the distress. Emotional regulation has been associated with numerous mental health disorders and has often been used to explain how specific conditions are related. In trauma-exposed firefighters, emotional regulation has shown a significant relationship with PTSD and depression [49]. As mentioned above, there is evidence linking PTSD symptom severity and the use of alcohol as a coping mechanism specifically in firefighters. Recent evidence suggests emotional regulation may explain the association between PTSD symptom severity and alcohol use as a coping mechanism in firefighters [116].

### 4.3. Distress Tolerance

Another factor that has shown promise with regards to the amelioration of mental health symptoms is distress tolerance. Distress tolerance is an individual difference trait that provides a self-report measure for an individual’s perceived capability to manage negative emotional states [117]. Lower distress tolerance scores have been associated with significant mental health concerns.

While previous research highlights the increased risk of suicide among firefighters exposed to greater occupational stress, the recent literature has found firefighters with higher distress tolerance attenuated this increased risk [55]. Further, due to increased occupational stress, firefighters are at a significantly greater risk to seek negative external coping mechanisms. Substance abuse has been reported by over half of professional firefighters [7]. However, it appears as though higher distress tolerance may reduce the risk of alcohol abuse among firefighters. In a sample of 652 trauma-exposed firefighters, distress tolerance was significantly related to alcohol abuse and other coping motives [118]. It follows logically that individuals with greater depressive symptomology experience negative emotional states more frequently. However, Yoon et al. [119] conducted a meta-analysis on distress tolerance and depression. Their results indicate a systematic relationship between lower distress tolerance and depression. That is, individuals with greater distress tolerance were less likely to display symptoms of depression. Distress tolerance has been negatively associated with numerous other mental health concerns, including PTSD [120] and anxiety [121].

Like emotional regulation, distress tolerance is associated with sleep quality. In a sample of military veterans, perceived sleep quality was positively associated with a self-reported distress tolerance with a poorer sleep relating to reduced distress tolerance as well as increased frustration in a distressing task [122]. Smith et al. [123] examined the association of sleep disturbance and distress tolerance in 652 firefighters and found that 48.6% had disturbed sleep and lower distress tolerance was significantly associated with greater sleep disturbance. To determine the best forms of treatment, the mechanisms linking distress tolerance, emotional regulation, and anxiety sensitivity to sleep quality warrant further investigation.

## 5. Sleep and Mental Health Interventions in Firefighters

### 5.1. Current Interventions

There is a need for interventions in full-time firefighters to determine applicable and successful strategies to improve their sleep quality and/or aspects of mental health. Table 1 summarizes the outcomes of various mental health and sleep interventions in full-time firefighters. Other intervention-based studies do exist; however, they are not specific to full-time firefighters but rather recruit firefighters (individuals employed by fire departments prior to becoming a full-time firefighter) or first responders (police, firefighters, EMS) [124,125,126]. It is important to specifically assess intervention outcomes for full-time firefighters as their schedule, trauma exposure, and occupational requirements are different than other first responders and recruit firefighters.

### 5.2. Suggested Intervention: Regular Exercise

Future interventional studies should assess the effects of regular exercise on both sleep quality and mental health in firefighters. A large amount of evidence exists that supports the efficacy of chronic exercise for improving subjective sleep quality in individuals suffering from insomnia symptoms. Multiple meta-analytic reviews have investigated exercise interventions on sleep quality and demonstrated overall significant improvements, especially in subjective sleep complaints [131,132,133,134,135]. In a meta-analysis on exercise as an alternative treatment for chronic insomnia, Passos et al. found that the efficacy of chronic exercise was similar to hypnotic drug use in improving symptoms of insomnia [131]. Multiple types of chronic exercise, from aerobic exercise to Tai Chi, have been shown to improve sleep [132]. Several mechanisms that provide a rationale for the improvement in insomnia symptoms from exercise have been suggested [133,136,137,138,139]; these include but are not limited to reducing core body temperature for sleep onset [140,141,142,143], increasing melatonin and serotonin secretion [131,144], reducing depression and anxiety, and improving autonomic regulation which may reduce hyperarousal associated with insomnia [143].

Exercise also has a strong history of alleviating symptoms of mental health disorders; it is often used as a stand-alone treatment method as well as in conjunction with traditional treatment methods for specific mental health conditions. A recent meta-analysis and systematic review support the use of aerobic exercise as an effective intervention (i.e., large or moderate-to-large effects) to alleviate the symptoms of depression in clinically depressed individuals [145]. Aylett et al. [146] conducted a systematic review and meta-analysis on exercise and anxiety. Their results indicate exercise to be effective at reducing anxiety in individuals diagnosed with anxiety disorders and individuals who self-report elevated anxiety. Additionally, the evidence suggests high-intensity exercise to be more effective at reducing anxiety, but both high- and low-intensity exercise were significantly more effective relative to no exercise [146]. While the effects of exercise on PTSD are still being explored, numerous studies highlight the positive benefits of exercise on PTSD. A recent review and meta-analysis have highlighted the beneficial effects of exercise on PTSD symptoms and some comorbid symptoms. Specifically, exercise reduced PTSD symptom severity and reduced substance abuse [147]. Vancampfort et al. [148] conducted a systematic review and meta-analysis on physical activity and suicidal ideation. While the results are based on cross-sectional data, there was a significant negative association between physical activity and suicidal ideation. Further, individuals who reported meeting current physical activity guidelines were significantly less likely to report suicidal ideation, while individuals who did not meet current physical activity guidelines were significantly more likely to report suicidal ideation [148].

While this is not a comprehensive list, the effects of exercise on mental health disorders, symptomology, and comorbid conditions have been well established. As mentioned above, anxiety, depression, PTSD, substance abuse, and suicidal ideation are often experienced together but remain independent of one another (i.e., not caused by each other). Implementing a single intervention to treat multiple mental health conditions may be challenging as each condition should be evaluated separately. However, exercise appears to have significant positive effects on all the mental health conditions described in this review.

## 6. Conclusions

Firefighters are at a greater risk for insomnia and mental health conditions due to their shift-working schedules, continual loud alerting emergency alarms, regular psychological and physical trauma exposure, and extreme occupational demands compared to the general population. In addition, sleep and mental health impairments are cyclical as both have been shown to affect the other. Therefore, it is of the utmost importance to determine the best strategies to improve these impairments in full-time firefighters in hopes of leading to a higher quality of life throughout their career as the community relies on their help. In addition, it is important for clinicians that work with firefighters, or other related occupations, to understand the cyclical relationship between insomnia and mental health conditions to better identify appropriate treatment strategies. Due to the supporting evidence, exercise as a treatment for insomnia and mental health conditions in firefighters should be further explored. Exercise interventions should assess multiple modes and intensities of exercise in addition to adherence rates to best determine the most effective and applicable type of regular exercise program to promote in fire departments.

## Figures and Tables

**Table 1:** Mental health and sleep interventions in full-time firefighters.

Study	Participants and Intervention	Outcome Measure(s)	Summary of Findings
Jang et al. (2020) [127], Korea	Participants: 39 firefighters Intervention: Single-group pre–post study design; utilized the Firefighter’s Therapy for Insomnia and Nightmares (FIT-IN) program which included two 90 min face-to-face group sessions and one 20 min individual phone session with a therapist for brief behavioral therapy for insomnia combined with imagery rehearsal.	•Insomnia severity index (ISI) •Disturbing dream and nightmare severity index (DDNSI) •Epworth Sleepiness Scale (ESS) •Depressive symptom inventory-suicidality subscale (DSI) •Patient Health Questionnaire-9 (PHQ-9) •Two-week sleep diary	The FIT-IN program resulted in a significant increase in sleep efficiency and decrease in sleep onset latency, the number of awakenings, and time in bed as well as insomnia and nightmare severity. Additionally, there were significant improvements in PTSD symptoms, depression, and daytime sleepiness.
Barger et al. (2016) [128], United States	Participants: 6101 firefighters Intervention: Firefighters were placed into a sleep health program (SHP) that provided a sleep health education training session with one of three delivery approaches: expert-led, train-the-trainer, or online.	•Screening for sleep disorders •Assessment of the sleep health educational material One-year follow-up on sleep	The expert-led delivery method of the SHP had the highest participation rate, highest assessment scores, and showed a greater number of firefighters seeking help for sleep disorders than the train-the-trainer and online delivery SHP approaches.
Mehrdad et al. (2015) [129], Iran	Participants: 27 firefighters with poor sleep quality Intervention: In a double-blind, randomized, placebo-controlled crossover trial, firefighters took 10 mg of zolpidem (sleeping pill) for two weeks and a placebo for two weeks with a two-week washout period between conditions.	•Persian version of the Pittsburgh Sleep Quality Index (PSQI)	There was a significant improvement in PSQI scores after the zolpidem period compared to the control period including improved sleep quality, sleep onset latency, sleep duration, habitual sleep efficiency, sleep disturbances, and daytime dysfunction.
Sullivan et al. (2017) [130], United States	Participants: 1189 firefighters Intervention: Firefighters attended a 30 min sleep health education training; those that attended the training were in the intervention group (n = 560), and those that did not attend the training were considered the control group (n = 629).	•Screening for sleep disorders •Firefighter health (sick time) and safety (motor vehicle crashes and injuries) were assessed from departmental records during the study’s two-week period •Baseline and end-of-year surveys on subjective improvements in sleep and general health	Firefighters that attended the sleep health education training were 24% less likely to file an official injury report during the study period than those in the control group. There were no changes in self-reported sleep, departmental injury, or motor vehicle crash rates between the groups during the study period.

## Data Availability

No new data were created or analyzed in this study. Data sharing is not applicable to this article.
